# Reduced Plasticity in Coupling Strength in the Aging SCN Clock as Revealed by Kuramoto Modeling

**DOI:** 10.1177/07487304231175191

**Published:** 2023-06-16

**Authors:** Anouk W. van Beurden, Janusz M. Meylahn, Stefan Achterhof, Renate Buijink, Anneke Olde Engberink, Stephan Michel, Johanna H. Meijer, Jos H. T. Rohling

**Affiliations:** * Department of Cell and Chemical Biology, Leiden University Medical Center, Leiden, The Netherlands; † Dutch Institute for Emergent Phenomena, University of Amsterdam, Amsterdam, The Netherlands; ‡ Department of Applied Mathematics, University of Twente, Enschede, The Netherlands; § Mathematical Institute, Leiden University, Leiden, The Netherlands

**Keywords:** circadian rhythm, suprachiasmatic nucleus, PERIOD 2, seasonal adaptation, aging, Kuramoto model, coupling strength

## Abstract

The mammalian circadian clock is located in the suprachiasmatic nucleus (SCN) and consists of a network of coupled neurons, which are entrained to the environmental light-dark cycle. The phase coherence of the neurons is plastic and driven by the duration of daylight. With aging, the capacity to behaviorally adapt to seasonal changes in photoperiod reduces. The mechanisms underlying photoperiodic adaptation are largely unknown, but are important to unravel for the development of novel interventions to improve the quality of life of the elderly. We analyzed the phase coherence of single-cell PERIOD2::LUCIFERASE (PER2::LUC) expression rhythms in the SCN of young and old mice entrained to either long or short photoperiod. The phase coherence was used as input to a 2-community noisy Kuramoto model to estimate the coupling strength between and within neuronal subpopulations. The model revealed a correlation between coupling strength and photoperiod-induced changes in the phase relationship among neurons, suggesting a functional link. We found that the SCN of young mice adapts in coupling strength over a large range, with weak coupling in long photoperiod (LP) and strong coupling in short photoperiod (SP). In aged mice, we also found weak coupling in LP, but a reduced capacity to reach strong coupling in SP. The inability to respond with an increase in coupling strength suggests that manipulation of photoperiod is not a suitable strategy to enhance clock function with aging. We conclude that the inability of aged mice to reach strong coupling contributes to deficits in behavioral adaptation to seasonal changes in photoperiod.

Many organisms increase their chance of survival and reproduction by anticipating seasonal changes in temperature and food availability. Internal clocks drive the circadian and seasonal rhythms responsible for physiological and behavioral adaptation. In mammals, the endogenous clock is located in the suprachiasmatic nucleus (SCN) of the anterior hypothalamus. The SCN is a relatively small structure that consists of approximately 20,000 neurons (Hastings et al., 2018). Generation of circadian rhythms occurs autonomously in all individual neurons and is based on a negative feedback loop between clock genes and their protein products (Welsh et al., 2010; Buijs et al., 2016; Hastings et al., 2019). This population of autonomous oscillators is able to produce a coherent rhythm of 24 h in electrical activity that acts as output of the SCN (Meijer et al., 2012; Herzog et al., 2017). The shape of this timing signal adapts to seasonal changes in photoperiod due to plasticity in phase coherence between the individual neurons. These changes in the phase coherence encode for the different seasons, reflecting day-length differences (VanderLeest et al., 2007; Ciarleglio et al., 2011; Buijink et al., 2016; Tackenberg and McMahon, 2018).

Although it is known that photoperiodic adaptation of the circadian clock is correlated with changes in phase relationship between SCN neurons, the mechanism is unknown. One possibility is that a decrease in coupling strength leads to a broadened phase distribution, when the day-length increases. Alternatively, phase differences can be driven by an active process, for example, due to repulsive coupling between subpopulations of SCN neurons (Myung et al., 2015). In such a scenario, the coupling strength could be equally strong in LP and SP. Subpopulations of SCN neurons form phase clusters that map approximately to the core and shell SCN (Foley et al., 2011; Evans et al., 2013; Buijink et al., 2016). The question addressed in this study is whether we can explain the changes in phase coherence between the neurons in different photoperiods by changes in coupling strength.

The coupling strength between neurons is largely determined by synaptic release of neurotransmitters and direct communication via gap junctions (Finger et al., 2020). We separately analyzed the coupling strength within and between neuronal subpopulations of the SCN. Based on neuropeptide expression, an anatomical subdivision can be made between the core and shell regions. Vasoactive intestinal polypeptide (VIP) and gastrin-releasing peptide (GRP) are primarily expressed in the core SCN, arginine vasopressin (AVP) in the shell SCN, and γ-aminobutyric acid (GABA) in almost all SCN neurons (Hegazi et al., 2019).

With aging, there is a reduction in peptidergic function, and there are significant changes in the GABAergic synaptic network of the SCN, as seen in a striking reduction of presynaptic terminals (Palomba et al., 2008). These alterations in the SCN network will cause reduced communication among neurons in the aged SCN (Nakamura et al., 2011; Farajnia et al., 2012). It has been shown that weakened circadian rhythmicity of the elderly have negative health effects and is causal to a broad array of diseases (Leng et al., 2019). Therefore, strengthening the clock in the aged is important, and strategies to do so rely on an identification of underlying mechanisms. One intervention to strengthen the clock could be to subject old mice to SP, because this may increase the phase coherence among the neurons in the aged SCN.

We used data from bioluminescence imaging of single-cell PERIOD2::LUCIFERASE (PER2::LUC) gene expression rhythms and Kuramoto models (Achterhof and Meylahn, 2021a, 2021b) to estimate the coupling strength within and between neuronal subpopulations in young and old mice entrained to long (LP, LD 16:8) and short (SP, LD 8:16) photoperiod (Buijink et al., 2016, 2020). Neuronal subpopulations of the SCN were identified with an unbiased clustering algorithm (Almog et al., 2019). We took into account that the coupling strengths are not the same within and between the different neuronal subpopulations, since it is known that in the SCN, the core projects densely to the shell while the shell projects only sparsely to the core (Welsh et al., 2010). The Kuramoto model predicted that coupling strength within and between subpopulations of SCN neurons contributes to photoperiod-induced changes in the phase relationship among neurons. We found that young animals can adapt their coupling strengths over a wide range. Therefore, young animals can easily adjust to both SP and LP. On the contrary, old animals have a diminished range over which they can adapt their coupling strengths, making it more difficult for them to adjust to SP.

## Materials and Methods

### Bioluminescence Imaging and Analysis

To obtain the parameters for the Kuramoto model, the PER2::LUC expression data from the studies (Buijink et al., 2016, 2020) were used. The dataset consisted of bioluminescence data from young (4-8 months) and old (22-28 months) homozygous PER2::LUC mice entrained to either LP (LD 16:8) or SP (LD 8:16). For details on the data collection, see Buijink et al. (2016). In short, mice were killed 1 to 3 h before lights-off. The brain was dissected, and the SCN was sliced in coronal slices with a VT 1000S vibrating microtome (Leica Microsystems, Wetzlar, Germany). Slices containing the SCN were visually identified and placed in a petri dish. The dish was transferred to a temperature-controlled (37 °C) light-tight chamber, equipped with an upright microscope and a cooled charge-coupled device camera (ORCA-UU-BT-1024, Hamamatsu Photonics Europe, Herrsching am Ammersee, Germany). Bioluminescence images were collected with a 1-h time resolution.

To analyze the time series of bioluminescence images, a custom-made MATLAB-based (Mathworks, Natick, MA, USA) program was used, as described in Buijink et al. (2016). Briefly, groups of 3 to 9 adjacent pixels with luminescence intensity above the noise level were defined as regions of interest (ROIs). Each ROI is referred to as a “single cell.” The average bioluminescence of all pixels in each ROI was calculated for the image series, which resulted in the bioluminescence traces representing PER2::LUC expression for all single-cell ROIs. For the analysis of rhythm characteristics, the raw PER2::LUC expression traces were smoothed and resampled to 1 data point per minute. Only single-cell traces containing at least 3 cycles with a period length between 20 and 28 h were included for further analysis.

The phase distribution and the Kuramoto order parameter (*r*) were calculated for all SCN slices. Phase distribution was defined as the SD of the peak times from all cells in a slice of the specified cycle in vitro. The order parameter is a measure for phase coherence and is based on the relative phase of the single cells. The order parameter was determined by first calculating the mean peak time $\left({\bar{t}}_{p}\right)$ of PER2::LUC expression of all cells (*j* *=* 1, . . ., *N*) for the specified cycle:

(1) $${\bar{t}}_{p}=\frac{\sum_{j=1}^{N}t_{p,j}}{N}.$$

Then the relative phase of each cell was approximated by first subtracting the peak time of the individual cell from the averaged peak time of all cells to get the relative peak time and then converting the relative peak time to its relative phase $(\theta_{r}):$

(2) $$\theta_{r,j}=\frac{\left({\bar{t}}_{p}-t_{p,j}\right)}{\tau }2\pi ,$$

where *τ* is the period in hours. The relative phase can be approximated because the sin(x) function is linear for small *x*, and the relative peak times are small in comparison with the period. Thereafter, the relative phase was transformed with Euler’s formula and the absolute value was taken to get the order parameter (*r*):

(3) $$r=\left|\frac{\sum_{j=1}^{N}e^{i\theta_{r,j}}}{N}\right|.$$

The order parameter can take values between 0 and 1, where 0 means that the neurons are completely unsynchronized and 1 means perfect synchrony.

### Community Detection

To identify functional clusters in the SCN neuronal network, we used a community detection method that was previously described by Almog et al. (2019). In brief, from the raw time series of PER2::LUC bioluminescence traces, a cross-correlation matrix was constructed. Next, with the use of random matrix theory, the global (SCN-wide) and local (neuron-specific) noise components were filtered out of the cross-correlation matrix. Clusters were detected with optimally contrasted functional signature, resulting in a positive overall correlation within clusters and a negative overall correlation between clusters, relative to the global SCN activity. Although the clustering algorithm was not bound to a pre-defined number of groups, the community detection method results consistently in 2 main groups of cells with a robust spatial distribution. The spatial distribution differed slightly for the anterior and posterior slices (Buijink et al., 2016, 2020). Hence, the resulting clusters were visually labeled as ventromedial and dorsolateral in the anterior SCN and as medial and lateral in the posterior SCN slices.

### Kuramoto Model

To model the SCN, we used a Kuramoto model. The Kuramoto model is a simple model that only contains phase information (Gu et al., 2019). First, we used a 1-community Kuramoto model to estimate the upper and lower bounds on the coupling strength and on the noise in the different experimental conditions. The noise term captures both the effect of the thermal environment in which the SCN resides (i.e., external noise) and the time-dependent variations in the natural frequencies of individual oscillators (see Rohling and Meylahn, 2020). The noise should be the same in all experimental conditions. With use of the 1-community model, we show that the amount of noise is indeed approximately the same in the different experimental conditions and therefore the differences in phase coherence are caused by changes in the coupling strength. Next, we extended our model to a 2-community Kuramoto model. By treating the noise as a constant factor in the 2-community Kuramoto model, we could separate the influence of the noise from the influence of the coupling strength on the phase coherence. We used the 2-community Kuramoto model to assess the relationship between the coupling strength within each subgroup and the coupling strength between the 2 subgroups.

The framework of the Kuramoto model we used in this study is extensively described in 4 recent papers (Garlaschelli et al., 2019; Meylahn, 2020; Achterhof and Meylahn, 2021a, 2021b). We will therefore not repeat all the steps involved, in detail, in the next 2 sections. However, we will show all main steps supported by references to the relevant parts of these papers.

### One-community Kuramoto Model

In the 1-community Kuramoto model, we consider one community of *N* oscillators. Each oscillator corresponds to a neuron in the SCN. The oscillators interact with a strength *K* which gives a mean-field interaction strength *K/N*. The phase angles of the oscillators are denoted by θ_*i*_, *i* *=* 1, . . ., *N* and represent the state of the neuron. The evolution of a single oscillator *i* is then given by,

(4) $$d\theta_{i}=\frac{K}{N}\sum_{j=1}^{N}sin\;(\theta_{j}-\theta_{i})\;dt+DdW_{t},$$

where *D* is the noise strength and *W_t_* is a standard Brownian motion. The model in equation (4) does not explicitly include the natural frequencies of the neurons or the external driving force of the light-dark cycle. This could be included explicitly, as done for the (noiseless) Kuramoto model in Childs and Strogatz (2008). The data we are considering, however, exhibit a number of properties that allow us to simplify the model as in equation (4). It is in steady-state and such that the period of the light-dark cycle, the average period of the neurons and the period associated with the average intrinsic frequency are approximately equal. This allows us to consider the system in the rotating frame of reference which matches the period of these quantities. As a result, we can set the average phase of the neurons, the average intrinsic frequency and the phase of the driving force to zero. Furthermore, whereas most circadian models assume that each neuron has a fixed intrinsic frequency, we assume that this internal frequency is not fixed, but probabilistic around a mean. This is due to the probabilistic nature of the processes in the transcriptional-translational feedback loop (Barkai and Leibler, 2000) and is shown experimentally by Herzog et al. (2004), where they show that dispersed neurons have a higher cycle-to-cycle variation in period than neurons that are connected in a network. The effect of the variation of intrinsic frequencies of each neuron can thus be included as a modification of the noise strength in the model. Finally, the driving forces in both LP and SPs have a period of 24 h; we note that as the period of the driving force matches that of the neurons in steady-state, we can include its effect as a modification of the interaction strength of the neurons. The parameters *D* and *K* in equation (4) are therefore modified parameters that include the distribution of intrinsic frequencies and driving force, respectively. Note that these simplifications would no longer hold if we were considering a driving force with a different period. Using these simplifying assumptions and the standard rewriting of the Kuramoto model, as given on page 39 of Sakaguchi (1988), the equation for a single representative neuron is given by,

(5) $$d\theta (t)=-Kr\;sin\;\theta (t)dt+DdW_{t}.$$

The noise can be understood as the effect of the thermal environment of the SCN and the time-dependent variations in the natural frequencies of individual oscillators. Now we will integrate the stochastic differential equation (SDE) in equation (5) from 0 to *T*, where we will take *T* to be 1 period of the average phase:

(6) $$\begin{array}{c} \Delta_{T}:=\theta (T)-\theta (0)\\ =-Kr\int_{0}^{T}sin\theta (s)ds+D\left(W_{T}-W_{0}\right), \end{array}$$

which, when taking the expectation, leads to,

(7) $$E\left[\Delta_{Τ}\right]=0.$$

Equation (6) allows us to relate 2 measurements of the phase of a single oscillator at different times to the noise parameter *D.* Since we are only interested in the change of phase ∆_*T*_ $\Delta_{T}$ between these 2 measurements, we can set the phase of the first measurement to zero, that is, θ(0)=0. Equation (7) then states that we expect the change in phase to be zero, which is a result of employing a rotating frame of reference. To derive upper and lower bounds for the noise strength parameter, we will first calculate bounds for the second moment of ∆_*T*_ $\Delta_{T}$ and solve these for *D*. To do this, note that we now have,

(8) $$E\left[\Delta_{T}^{2}\right]=E\left[\theta {\left(T\right)}^{2}\right],$$

and by Itô’s lemma in its integral form, we have that,

(9) $$f\left(\theta (T)\right)=f\left(\theta (0)\right)+\int_{0}^{T}\frac{df}{d\theta }d\theta (t)+\frac{1}{2}\int_{0}^{T}\frac{d^{2}f}{d\theta^{2}}dt.$$

Plugging in dθ(t), as given by equation (5), leads to,

(10) $$\theta {\left(T\right)}^{2}=-2Kr\int_{0}^{T}\theta (s)\;sin\;\theta (s)ds+2D\int_{0}^{T}\theta (s)dW_{s}+TD^{2}.$$

The samples of the phase difference in subsequent cycles show that $\Delta_{T}$ is much smaller than 1. This allows us to employ a Taylor expansion of the sine function in the first integral of equation (10). Since this expansion is alternating and has higher order terms smaller than lower order terms, we can find an upper bound for the second moment of $\Delta_{T}$ by truncating the expansion at a negative term, and find a lower bound by truncating the expansion at a positive term (note the reversal here due to the sign of the integral containing sine in equation (10)). Taking $sinx=x-(x^{3}/3!)$ gives,

(11) $$\begin{array}{l} E\left[\Delta_{T}^{2}\right]=D^{2}T-2Kr\\ \int_{0}^{T}E\left[\left(\theta {\left(s\right)}^{2}-\frac{\theta {\left(s\right)}^{4}}{3!}+o(\theta {\left(s\right)}^{6})\right)\right]\;ds. \end{array}$$

As explained above, using only the first term of the expansion implies that,

(12) $$E\left[\Delta_{T}^{2}\right]\geq D^{2}T-2Kr\int_{0}^{T}E\left[\Delta_{s}^{2}\right]\;ds.$$

Since we are in stationarity, this gives an upper bound for the noise strength (*D_+_*),

(13) $$D^{2}\leq \frac{E\left[\Delta_{T}^{2}\right]}{T}(1+2KrT)=:D_{+}^{2}.$$

Using one more term in the expansion for sine gives,

(14) $$\begin{array}{l} E\left[\Delta_{T}^{2}\right]\leq D^{2}T-2Kr\int_{0}^{T}E\left[\theta {\left(s\right)}^{2}-\frac{\theta {\left(s\right)}^{4}}{3!}\right]\;ds\\ =D^{2}T-2KrTE\left[\Delta_{T}^{2}\right]+Kr\frac{E\left[\Delta_{T}^{4}\right]}{3!}, \end{array}$$

so that the noise strength is bounded from below (*D_–_*) by,

(15) $$D^{2}\geq \frac{E\left[\Delta_{T}^{2}\right]}{T}(1+2KrT)-2Kr\frac{E\left[\Delta_{T}^{4}\right]}{3!}=:{D^{2}}_{-}.$$

Since we have experimentally obtained time series data, we are able to numerically calculate upper and lower bounds for the noise strength in terms of the interaction strength *K*. This holds in the case that sine is approximated well by the expansion used, which we posit to be the case since the spread of the phases around the average is small relative to the size of the entire cycle.

To do this, we need unbiased estimators of the second and fourth moments. Since the mean is zero, the fourth moment is equal to the fourth central moment for which an unbiased estimator is given by the fourth *h*-statistic:

(16) $$h_{4}=\frac{3(3-2n)n^{2}m_{2}^{2}+n^{2}(n^{2}-2n+3n)m_{4}}{(n-3)(n-2)(n-1)n},$$

where *n* is the sample size and *m_p_* is the *p*th sample central moment given by,

(17) $$m_{p}:=\frac{1}{n}\sum_{i=1}^{n}{\left(x_{i}-m\right)}^{p},$$

with *m* the sample mean. An unbiased estimator for the variance is,

(18) $$h_{2}=\frac{nm_{2}}{\left(n-1\right)}.$$

Now, if we want to calculate the interaction strength parameter *K* for a single community, we must solve the equation,

(19) V(Cr)=r,

for *C*, where,

(20) $$V(x)=\frac{BesselI\left[1,x\right]}{BesselI\left[0,x\right]},$$

and BesselI[0,x]andBesselI[1,x] are modified Bessel functions of the first kind. Note that the function V(.) has the following properties: (1) V(0)=0, (2) $\underset{x\to \infty }{\lim}V(x)=1$ , and (3) V(.) is monotonically increasing, so that there is a unique non-zero solution to equation (19) for any 0≤r<1. Equation (19) is derived in the general case with natural frequencies in equation (1.21) of Garlaschelli et al. (2019). In the case with all ω=0, it simplifies to equation (19), as shown in equation (3.51) of Garlaschelli et al. (2019). From the bioluminescence time series data, we have calculated *r* so that we can use numerical methods (like FindRoot in MATHEMATICA) to solve for *C*. In the 1-community model, C=2K/D , so that we can find upper and lower bounds for *K*:

(21) $$\frac{CD_{-}}{2}\leq K\leq \frac{CD_{+}}{2}.$$

Now both *D_-_* and *D_+_* depend on *K* so that we find,

(22) $$K_{-}\leq K\leq K_{+},$$

with

(23) $$K_{-}:=\frac{1}{24}\left(\begin{array}{l} C^{2}(6h_{2}-h_{4})r\\ +\sqrt{C^{4}{\left(h_{4}-6h_{2}\right)}^{2}r^{2}+\frac{144C^{2}h_{2}}{T}} \end{array}\right),$$

and

(24) $$K_{+}:=\frac{1}{4}\left(C^{2}h_{2}r+\sqrt{\frac{4C^{2}h_{2}+C^{4}h_{2}^{2}r^{2}T}{T}}\right).$$

To recapitulate, we calculated the order parameter from the bioluminescence data. Next, we used the order parameter to solve the non-linear equation (20) for *C*, and then we inferred the range for the coupling strength using the order parameter, *C*, and the second and fourth moment.

### Two-community Kuramoto Model

The 1-community Kuramoto model was expanded to a 2-community model (Meylahn, 2020; Achterhof and Meylahn, 2021a, 2021b) for which each community consists of *N* oscillators. The oscillators in the same community interact with strength *K*, and oscillators in different communities interact with strength *L*. The phase angles of the oscillators in the first community are denoted by θ_1_,*_i_, i* *=* 1, . . ., *N* and in the second community by θ_2_,*_j_, j* *=* 1, . . ., *N*. The equations governing their evolution are,

(25) $$\begin{array}{l} d\theta_{1,i}(t)=\frac{K_{1}}{2N}\sum_{K=1}^{N}sin\left(\theta_{1,k}-\theta_{1,i}(t)\right)dt\\ +\frac{L_{1}}{2N}\sum_{l=1}^{N}sin\left(\theta_{2,l}-\theta_{1,i}(t)\right)dt\\ +\sqrt{D}dW_{1,i}(t), \end{array}$$

and

(26) $$\begin{array}{l} d\theta_{2,j}(t)=\frac{K_{2}}{2N}\;\sum_{l=1}^{N}sin\left(\theta_{2,l}-\theta_{2,j}(t)\right)dt\\ +\frac{L_{2}}{2N}\;\sum_{k=1}^{N}sin\left(\theta_{1,k}-\theta_{2,j}(t)\right)dt\\ +\sqrt{D}dW_{2,j}(t). \end{array}$$

In the 1-community Kuramoto model, we found that *D* does not depend on the phase coherence and that *D* is close to 1 for all experimental conditions. Therefore, we take *D* *=* 1 in the 2-community Kuramoto model. As we are only interested in the qualitative relationship between the synchronization and interaction strengths, we could set it to any positive constant. Furthermore, we made the assumption that the average phase is the same in both communities (i.e., ψ_1_ *=* ψ_2_ *=* 0). Now we can calculate the relationship between *K*_1_ and *L*_1_ and between *K*_2_ and *L*_2_ in the infinite oscillator limit by solving the equations,

(27) $$V\left(\frac{K_{1}r_{1}+L_{1}r_{2}}{D}\right)=r_{1},$$

and

(28) $$V\left(\frac{K_{2}r_{2}+L_{2}r_{1}}{D}\right)=r_{2},$$

which are stated in Proposition II.6 and Remark II.8 of Achterhof and Meylahn (2021a) and have been derived in Appendix A1 of the same article. In the above equations, *K*_1_ and *K*_2_ represent the coupling strengths within, respectively, subpopulations 1 and 2. *L*_1_ and *L*_2_ represent the interaction strength between subpopulations, where *L*_1_ is the strength from subpopulation 2 to subpopulation 1 and *L*_2_ is the strength from subpopulation 1 to subpopulation 2. *r*_1_ and *r*_2_ are the order parameters in, respectively, subpopulations 1 and 2. The function *V*(*·*) is the same function as in equation (20), so there is a unique non-zero solution to the equation,

(29) $$r_{1}=V\left(C_{1}\right),$$

for *C*_1_ (Meylahn, 2020), with $C_{1}=K_{1}r_{1}+L_{1}r_{2}.$ We therefore must have,

(30) $$K_{1}=\frac{C_{1}}{r_{1}}-\frac{r_{2}}{r_{1}}L_{1}.$$

We can do the same for equation (28).

### Coupling Strength Analysis

With the 2-community model, we found a linear relationship between the coupling strength within a subpopulation and the interaction strength between subpopulations. We created a search-space with range [0: 10] for *K*_1_ and *K*_2_ and range [–5: 10] for *L*_1_ and *L*_2_. Here, a negative coupling strength indicates repulsive coupling. We investigated the search-space of the lines with 2 different approaches to determine whether there are differences in the range over which young and old mice can adapt their coupling strengths between photoperiods and whether the differences in coupling strengths between young and old mice are larger in LP or SP.

For the first approach, we investigated all possible solutions in the search-space, where each pair of values for (*K*_1_, *L*_1_) and (*K*_2_, *L*_2_) located on the linear line is a possible solution. We used an interval of 0.1 for *K* and numerically calculated the corresponding value for *L*. To compare the coupling strengths between the experimental conditions, we defined the total adaptive capacity as, ∆*K*_1_ +∆|*L*_1_| + ∆*K*_2_ +∆|*L*_2_|. $\Delta K_{1}+\Delta \left|L_{1}\right|+\Delta K_{2}+\Delta \left|L_{2}\right|.\Delta$

**Figure 1.**
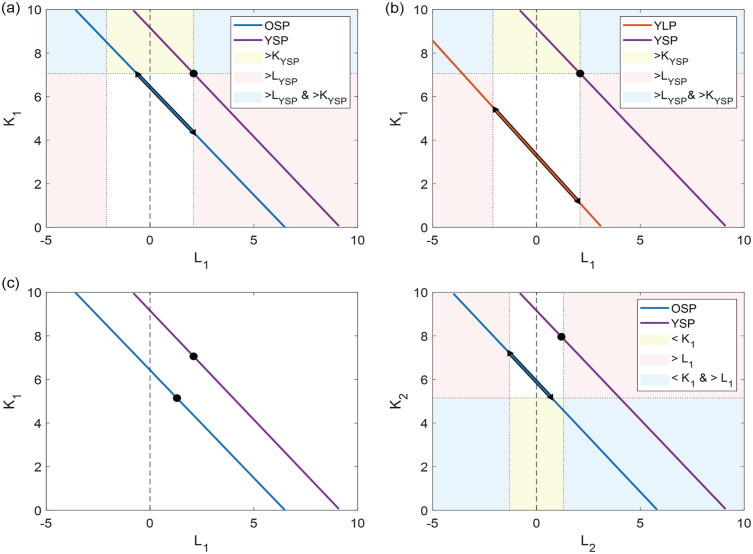
Illustration of constraints. Example of the (a) first and (b) second constraint. The black marked areas on the lines indicate the possible range of values for OSP and YLP based on the reference point for YSP for the first and second constraints, respectively. The colored background indicates the regions where either *K* is larger (yellow), *L* is larger (pink), or both *K* and *L* are larger (blue). (c) Example of the third constraint. The black marked area indicates the possible range of values for OSP based on the reference points for YSP and OSP. The colored background indicates where either *K*_1_ < *K*_2_ (yellow), *L*_1_ > *L*_2_ (pink), or both *K*_1_ < *K*_2_ and *L*_1_ > *L*_2_ (blue). Abbreviations: OSP = old short photoperiod; YLP = young long photoperiod; YSP = young short photoperiod. Color version of the figure is available online.

For the second approach, we added 3 constraints to the search-space and investigated the remaining solutions. Combinations of value pairs for (*K*_1_, *L*_1_) and (*K*_2_, *L*_2_) were only included (1) when *K* and *L* are higher in young animals than in old animals (i.e., *K*_1*young*_ *>* *K*_1*old*_, *K*_2*young*_ *>* *K*_2*old*_, *|L*_1_*|_young_* *> |L*_1_*|_old_*, and *|L*_2_*|_young_* *> |L*_2_*|_old_*), (2) when *K* and *L* are higher in SP than LP (i.e., *K*_1*SP*_ *>* *K*_1_*_LP_, K*_2*SP*_ *>* *K*_2_*_LP_, |L*_1_*|_SP_* *> |L*_1_*|_LP_*, and *|L*_2_*|_SP_* *> |L*_2_*|_LP_*), and (3) when the relationship between *K*_1_ and *K*_2_ and between *L*_1_ and *L*_2_ is in the same direction for the different experimental conditions (e.g., when *K*_1*YSP*_ *>* *K*_2*YSP*_ then *K*_1*YLP*_ *>* *K*_2*YLP*_, *K*_1*OSP*_ *>* *K*_2*OSP*_, and *K*_1*OLP*_ *>* *K*_2*OLP*_). The constraints are illustrated in Figure 1.

## Results

### Synchronization of PER2::LUC Rhythms in the SCN

**Figure 2.**
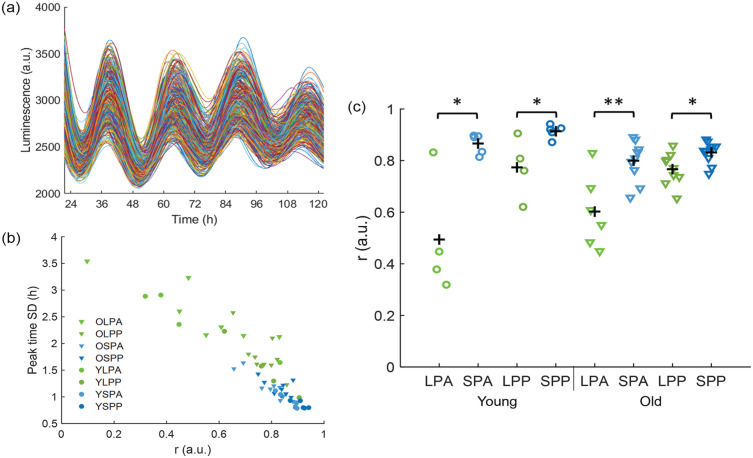
Synchronization of the SCN. (a) Example of smoothed intensity traces of PER2::LUC expression from single cells in the anterior SCN of a young mouse in SP. (b) Pearson correlation between *r* and peak time dispersion for all recordings (*n* = 54, *R* = –0.91). (c) The order parameter *r* is calculated for all slices and is shown for anterior and posterior slices in LP (LPA and LPP, respectively, green) and SP (SPA and SPP, respectively, blue) in young (circles) and old (triangles) mice. The black crosses indicate the mean. Abbreviations: SCN = suprachiasmatic nucleus; PER2::LUC = PERIOD2::LUCIFERASE; SPA = short photoperiod anterior; SPP = short photoperiod posterior; LPA = long photoperiod anterior; LPP = long photoperiod posterior. Color version of the figure is available online. *p < 0.05. **p < 0.01.

We calculated the order parameter (*r*), using equations (1) to (3), and peak time dispersion from the smoothed bioluminescence traces (Figure 2a) for all SCN slices in the different experimental conditions. To test whether the order parameter is an appropriate measure for phase coherence, we calculated the Pearson correlation coefficient between *r* and peak time dispersion, which was taken as a measure for phase coherence in previous studies (Buijink et al., 2016, 2020). The correlation coefficient showed a strong negative correlation between *r* and peak time dispersion (*R* = –0.91; Figure 2b), which is as we expected, as high dispersion should lead to lower phase coherence (*r*). Then, we compared the values of *r* between the different experimental conditions. Independent *t* tests showed that the *r* value was always significantly higher in SP than in LP in both young and old mice (young anterior, LP: 0.49 ± 0.23, *n* = 4, young anterior, SP: 0.87 ± 0.04, *n* = 5, *p* < 0.05; young posterior, LP: 0.77 ± 0.12, *n* = 4, young posterior, SP: 0.91 ± 0.03, *n* = 5, *p* < 0.05; old anterior, LP: 0.53 ± 0.23, *n* = 7, old anterior, SP: 0.80 ± 0.08, *n* = 10, *p* < 0.01; old posterior, LP: 0.77 ± 0.06, *n* = 9, old posterior, SP: 0.83 ± 0.04, *n* = 10, *p* < 0.05; Figure 2c). We refer to the studies of Buijink et al. (2016, 2020) for a more comprehensive analysis on the rhythm characteristics of the data.

### Coupling Strength and Noise Estimation

**Figure 3.**
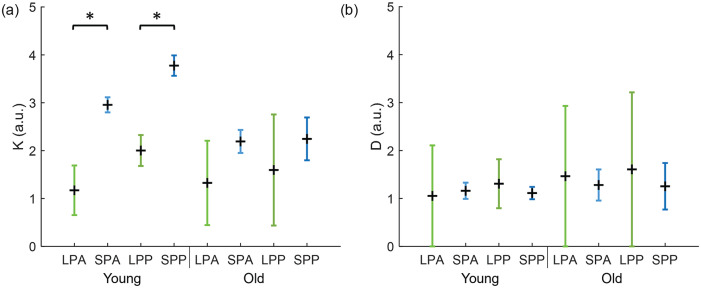
Range of *K* and *D* in different experimental conditions. (a) Range for the coupling strength between neurons in anterior and posterior slices in long (LPA and LPP, respectively, green) and short (SPA and SPP, respectively, blue) photoperiod in young and old mice. The range is based on the distance between the median of the upper and lower bound of *K* in each condition. The black cross indicates the mean of the range. (b) Range for the noise term in anterior and posterior slices in long (LPA and LPP, respectively, green) and short (SPA and SPP, respectively, blue) photoperiod in young and old mice. The range is based on the distance between the median of the upper and lower bound of *D* in each condition. The black cross indicates the mean of the range. Abbreviations: short photoperiod posterior; SPA = short photoperiod anterior; SPP = short photoperiod posterior; LPA = long photoperiod anterior; LPP = long photoperiod posterior. Color version of the figure is available online.

We used the order parameter (*r*), as calculated from the bioluminescence traces, to estimate the coupling strength (*K*) between the neurons in the SCN and to estimate the amount of noise (*D*) in the different experimental conditions. The noise represents the thermal environment of the SCN (see “Materials and Methods”). For both the coupling strength and the noise, we calculated for each slice an upper and lower bound (Suppl. Fig. S1). A 1-sample Kolmogorov-Smirnov test showed that *K* and *D* were not normally distributed (*p* > 0.05). To compare the bounds of *K* and *D* between the experimental conditions, we used non-parametric independent-samples median tests. The lower and upper bound of *K* is always significantly higher in SP than LP (*p* < 0.05), except for the upper bound of the posterior SCN in old mice (Suppl. Fig. S1A; *p* > 0.05). There were no significant differences in the lower and upper bound of *D* between the experimental conditions (Suppl. Fig. S1B; *p* > 0.05). Next, the ranges between the medians of the upper and lower bounds for *K* and *D* in the different experimental conditions were calculated (Figure 3). The ranges for *K* and *D* only differ significantly between conditions when the mean values of the ranges that are compared are not situated within each other’s range. Therefore, the coupling strength is definitely higher in SP than LP in young mice (*p* < 0.05). This is in agreement with Buijink et al. (2016). For old mice, the differences in coupling strength between SP and LP are not significant (*p* > 0.05), as the mean value of the range in SP is within the range of LP. The range between the upper and lower bound for *D* is larger for LP than SP in both young and old mice; however, the range does not differ significantly between the experimental conditions (*p* > 0.05). The mean value between the upper bound and lower bound of *D* is close to 1 for all experimental conditions. This shows that the noise will not affect the results of the 2-community Kuramoto model, as *D* has a constant value that is independent of the phase coherence.

### Synchronization of the Neuronal Subpopulations

**Figure 4.**
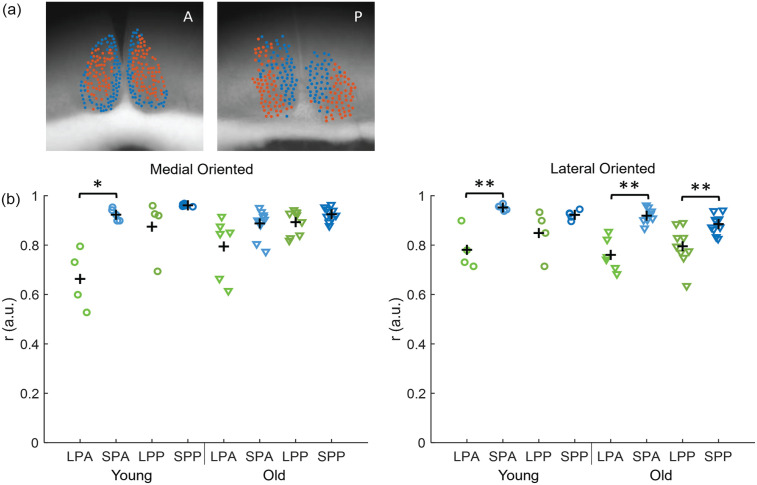
Synchronization in the SCN neuronal subpopulations. (a) Examples of the projection of cell location on bright field image of an anterior (A, left) and posterior (P, right) SCN of a young animal. The blue cells represent the medial-oriented cluster and the orange cells the lateral-oriented cluster. (b) The order parameter is calculated for the medial-oriented (left) and lateral-oriented (right) neuronal subpopulations in all slices and is shown for anterior and posterior slices in LP (LPA and LPP, respectively, green) and SP (SPA and SPP, respectively, blue) in young (circles) and old mice (triangles). The black crosses indicate the mean of the experimental condition. Abbreviations: SCN = suprachiasmatic nucleus; short photoperiod posterior; SPA = short photoperiod anterior; SPP = short photoperiod posterior; LPA = long photoperiod anterior; LPP = long photoperiod posterior. Color version of the figure is available online. *p < 0.05. **p < 0.01.

Next, we identified neuronal subpopulations within the SCN using an unbiased community detection algorithm (Almog et al., 2019). The community detection algorithm resulted consistently in 2 main groups of cells with a robust spatial distribution, without prespecifying the number of groups. The spatial distribution of the neuronal subpopulations corresponded only partially with the division of the SCN in dorsomedial (shell) and ventrolateral (core) SCN based on neuropeptide content (Yan et al., 2007) and differed slightly between the anterior and posterior slices (Figure 4a). From now on, we will refer to the ventromedial cluster from anterior slices and the medial cluster from posterior slices as the *medially oriented cluster.* We will refer to the dorsolateral cluster from anterior slices and the lateral cluster from posterior slices as the *laterally oriented cluster* for simplicity. Note that we used the same clustering of the data as reported in Buijink et al. (2016, 2020). Hence, we refer to these studies for detailed analysis on the community structure.

We calculated the order parameter for the bioluminescence traces for each subpopulation. Paired-sampled *t* tests showed that *r* was always significantly higher in each of the neuronal subpopulations compared with the SCN as a whole (*p* < 0.05, Figures 2c and 4b). For the medially oriented cluster, there was only a significant difference in *r* between LP and SP in the anterior SCN of young mice (young anterior, LP: 0.66 ± 0.12, *n* = 4, young anterior, SP: 0.92 ± 0.02, *n* = 5, *p* < 0.05; Figure 4b). For the laterally oriented cluster, *r* was significantly higher in SP than in LP in nearly all conditions, except for the posterior SCN of young mice (young anterior, LP: 0.78 ± 0.08, *n* = 4, young anterior, SP: 0.95 ± 0.01, *n* = 5, *p* < 0.01; young posterior, LP: 0.85 ± 0.09, *n* = 4, young posterior, SP: 0.92 ± 0.02, *n* = 5, *p* = 0.286; old anterior, LP: 0.74 ± 0.08, *n* = 7, old anterior, SP: 0.92 ± 0.03, *n* = 10, *p* < 0.01; old posterior, LP: 0.80 ± 0.08, *n* = 9, old posterior, SP: 0.89 ± 0.04, *n* = 10, *p* < 0.01; Figure 4b).

### Estimating Coupling Strength Within and Between Communities

**Figure 5.**
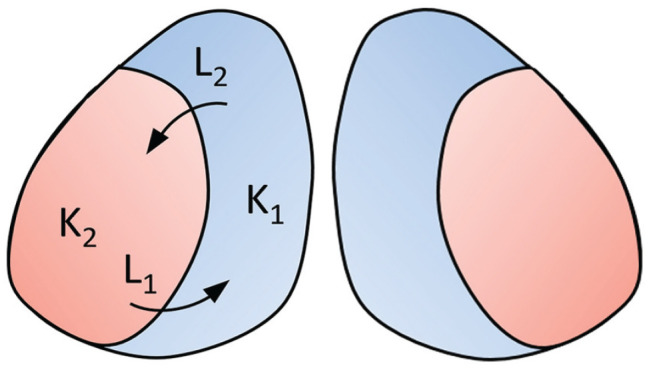
Simplified representation of the 2-community Kuramoto model. The blue area represents the medial-oriented cluster in which the coupling strength is denoted by *K*_1_ and the orange area represents the lateral-oriented cluster in which the coupling strength is denoted by *K*_2_. *L*_1_ shows the interaction strength from the lateral-oriented cluster to the medial-oriented cluster, and *L*_2_ shows the interaction strength from the medial-oriented cluster to the lateral-oriented cluster.

Next, we calculated the averaged order parameters for the neuronal subpopulations in the different experimental conditions. Here we took the anterior and posterior slices within the same experimental condition together, because the 2-community Kuramoto model only allows for 2 communities (i.e., the medial- and lateral-oriented clusters). The resulting order parameters for the medial-oriented cluster were *r* = 0.77 for young mice in LP, *r* = 0.94 for young mice in SP, *r* = 0.84 for old mice in LP, and *r* = 0.91 for old mice in SP. And for the lateral-oriented cluster, the resulting order parameters were *r* = 0.81 for young mice is LP, *r* = 0.94 for young mice in SP, *r* = 0.77 for old mice in LP and *r* = 0.90 for old mice in SP. We used the averaged order parameters, as computed from the bioluminescence traces, to estimate the coupling strength within and between the neuronal subpopulations in the SCN. Figure 5 shows a simplified representation of the model. We made the assumption that *D* *=* 1 for all experimental conditions, since the changes in *D* were minor in the results of the 1-community Kuramoto model. To find the relationship between *K*_1_ and *L*_1_ and between *K*_2_ and *L*_2_, equations (27) and (28) (“Materials and Methods” section), which are derived from the extended Kuramoto model (Achterhof and Meylahn, 2021a, 2021b) were numerically solved. *K*_1_ represents the coupling strength within the medial-oriented cluster and *K*_2_ the coupling strength within the lateral-oriented cluster. *L*_1_ represents the interaction strength from the lateral-oriented cluster to the medial-oriented cluster and *L*_2_ the interaction strength from the medial-oriented cluster to the lateral-oriented cluster, and *r*_1_ is the order parameter for the medial-oriented cluster and *r*_2_ is the order parameter for the lateral-oriented cluster.

**Figure 6.**
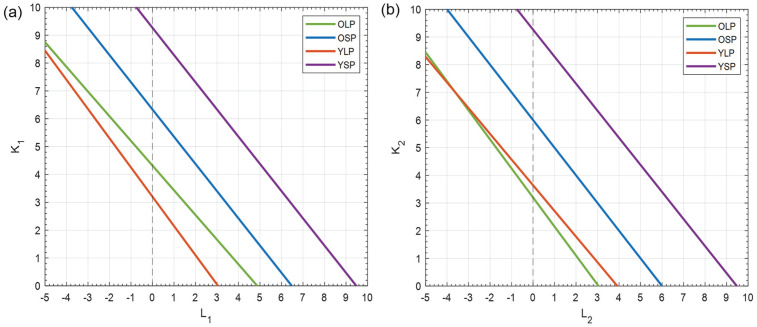
Coupling strength within and between neuronal subpopulations of the SCN. (a) The relation between the coupling strength (*K*_1_) within the medial-oriented cluster and the interaction strength (*L*_1_) from the lateral-oriented cluster to the medial-oriented cluster are shown for the different experimental conditions. The green line indicates old mice in LP; the blue line, old mice in SP; the orange line, young mice in LP; and the purple line, young mice in SP. There is a range of values for *K*_1_ and *L*_1_ that result in the same synchronization as observed in the bioluminescence data. (b) The same as (a) for the coupling strength (*K*_2_) within the lateral-oriented cluster and the interaction strength (*L*_2_) from the medial-oriented cluster to the lateral-oriented cluster. Abbreviation: SCN = suprachiasmatic nucleus. Color version of the figure is available online.

In Figure 6, the relationship between *K* and *L* is shown for the different experimental conditions. For both subpopulations, we found a negative linear relation between *K* and *L*. The coupling strength (*K*) within a neuronal subpopulation is always positive, and the interaction strength (*L*) between the neuronal subpopulations can be both positive or negative, where a negative strength indicates repulsive coupling.

Since the relation between *K*_1_ and *L*_1_ appeared to be linear, we can express each line as,

(31) $$K_{1}=a_{1}L_{1}+b_{1},$$

in which $a_{1}=-r_{2}/r_{1}$ and *b*_1_ is positively dependent on *r*_1_. The relationship between *K*_2_ and *L*_2_ can be described in the same way, by interchanging the role of *r*_1_ and *r*_2_. From the relations between the lines, we can investigate how well young and old mice can adapt to different photoperiods.

From our available experimental data, it is not possible to obtain precise values for *K*_1_, *K*_2_, *L*_1_, and *L*_2_. We know that the values for *K*_1_, *K*_2_, *L*_1_, and *L*_2_ are located somewhere on the lines (Figure 6), but we do not know the exact spot on the lines. However, we can investigate the search-space of the lines to determine (1) whether there are differences in the range over which young and old mice can adapt their coupling strengths between photoperiods and (2) whether the differences in coupling strengths between young and old mice are larger in LP or SP. The adaptation capacity to photoperiods is deducted from the degree of variation in coupling within and between clusters in different conditions. Analysis of the search-space revealed that only in ~32% of all possible solutions, old mice have higher adaptive capacity than young mice and that in ~37% of the solutions, the differences in coupling strength between young and old mice are larger in LP than SP.

Note that this information was derived from all possible solutions in the search-space, including solutions that are unlikely to be present in real life. Therefore, we added 3 constraints and investigated the search-space of the lines again. Spots on the lines for *K*_1_, *K*_2_, *L*_1_, and *L*_2_ were only included (1) when *K* and *L* are higher in young animals than in old animals, (2) when *K* and *L* are higher in SP than LP, and (3) when the relationship between *K*_1_ and *K*_2_ and between *L*_1_ and *L*_2_ is in the same direction for the different experimental conditions. Then, in less than 0.005% of the remaining solutions do old mice have a higher total adaptive capacity than young mice. This indicates that the range over which young mice can adapt their coupling strength is larger than the range over which old animals can adapt their coupling strength. Furthermore, in only 9.0% of the remaining solutions, the differences in coupling strength between young and old mice were larger in LP than SP.

## Discussion

In this study, we analyzed single-cell PER2::LUC gene expression rhythms of SCN neurons to determine the phase coherence between neurons in the SCN and in neuronal subpopulations of the SCN. By use of the 1-community Kuramoto model, we found that the coupling strength between SCN neurons is stronger in SP than LP. Next, we expanded to a 2-community Kuramoto model, which revealed a negative linear relationship between the coupling strength within a subpopulation and the coupling strength the subpopulations experience from the other subpopulation. Furthermore, we found evidence that the SCN of old animals is less capable of adjusting to SP because of an inability to respond to SP with an increase in coupling strength. There is less of a difference in coupling strength between young and old animals in a LP, when only a low degree of coupling is required.

In 2 other recent studies, a model similar to our model was used. Hannay et al. (2020) used the Ott-Antonsen ansatz to investigate the processing of light information in the SCN, and Goltsev et al. (2022) used a reduced Kuramoto model to investigate the dynamical behavior of the core and shell SCN under different lighting conditions. Their results showed that the 2-community Kuramoto model captures essential features of phase coherence in the SCN. This validates our method to use the phase coherence, as calculated from empirical data, to estimate the coupling strength between and within subpopulations of the SCN with a 2-community Kuramoto model.

In our study, the 2-community Kuramoto model appeared to be highly suitable to determine network properties of the SCN that are not directly measurable but can be derived on the basis of available empirical data. For example, the model made clear that while the differences in phase coherence between young and old animals are approximately the same in SP and LP, the differences in coupling strength between young and old animals are larger in SP than in LP. PER2::LUC time traces of single cells seemed suitable to determine the coupling strengths between and within neuronal subpopulations of the SCN. Although we could not obtain the exact values for *K*_1_, *K*_2_, *L*_1_, and *L*_2_, we could narrow down the number of possible solutions by adding biologically based constraints. The 2-community Kuramoto model is highly suitable for adding constraints because of its unique property that the coupling strengths between and within the 2 communities do not have to be the same.

The constraints we added were based on neurotransmitter expression within the SCN together with our results from the 1-community Kuramoto model. We assumed the coupling strengths would be stronger in young animals than in old animals based on reductions in the synaptic network and changes in membrane properties, leading to altered neurotransmission in the aged SCN (Palomba et al., 2008; Farajnia et al., 2012; Leise et al., 2013). Second, we assumed the coupling strengths would be stronger in SP than LP, because the 1-community model showed that the differences in synchronization between photoperiods were caused by differences in coupling strength and were not due to more or less noise in the system. And finally, we assumed the relationship between the coupling strengths to be in the same direction between different experimental conditions. This constraint is based on the fact that VIP, which is an important neurotransmitter for synchronizing SCN neurons, is only expressed in the ventral (or core) SCN (Hegazi et al., 2019; Finger et al., 2020). Furthermore, it is known that the dorsal SCN receives strong input from the ventral SCN, whereas the ventral SCN receives sparse input from the dorsal SCN (Taylor et al., 2017). However, since our clusters only partially overlap with neuropeptide content in the SCN, we decided not to specify whether *K*_1_ and *L*_1_ should be higher than *K*_2_ and *L*_2_, or the other way around. Identifying more constraints on the coupling strength between (and within) communities could help in narrowing down the search-space, so that we can better understand the mechanism of coupling in the SCN under different conditions.

Adding the above-mentioned constraints to the model revealed that young mice can adapt their coupling strengths over a larger range than old mice, which suggests that the SCN of young mice has larger adaptive capacity than the SCN of aged mice. It furthermore revealed that the differences in coupling strength between young and old mice are larger in SP than in LP in 91% of the possible solutions. This suggests that it is more difficult for old mice to adjust to SP than to LP. Although the effects of a reduced range of coupling strengths in old mice seem negligible at the molecular level, these results are in agreement with previously reported effects of aging downstream of the SCN (Buijink et al., 2020). Buijink et al. showed that old mice had a reduced rhythm amplitude in behavior and that old mice particularly had a strongly reduced ability to adapt to SP behaviorally. Hence, exposure to SP is not a useful intervention for boosting the rhythm of old animals. However, when interpreting the results, we need to keep in mind that the coupling strengths are inferred from the model. There are possibly other factors, such as reduction in the strength of photic input or increased variance in the intrinsic periods of the SCN oscillators, that could contribute to less phase coherence in older mice.

Previous modeling work by Myung and Pauls (2018) also used a Kuramoto model to describe the interaction between functional oscillators in the SCN to encode for seasonal time. Their work pioneered in showing the existence of repulsive coupling from the ventral part of the SCN to the dorsal part of the SCN and attractive coupling from the dorsal part of the SCN to the ventral part of the SCN. They suggested that there is an increase in repulsive coupling from SP to LP, creating a wider peak time dispersion between neurons in LP. Their framework fits nicely within our model where we added additional parameters for the coupling strength within subpopulations of neurons in the SCN.

Besides repulsive coupling, a broadened peak time dispersion between neurons in LP could be caused by a reduction in the coupling strength. From the relationship between the order parameter and the coupling strength, we know that the coupling strength increases when the order parameter increases. This would suggest a reduction in coupling strength is the correct mechanism. However, we were not able to perform measurements within the neuronal subpopulations, without one subpopulation being influenced by the other. Therefore, we do not know whether the phase coherence of the subpopulations of the SCN differs from the phase coherence measured over the entire SCN, due to changes in coupling strength within the clusters or due to the interaction strength between the clusters. As a result, it is impossible to determine which mechanism is the correct one from our data and analysis, and we need to rely on constraints to interpret the results of our model.

We used the order parameter of the Kuramoto model as a measure for neuronal synchronization within the SCN. The order parameter was normalized to obtain a value between 0 and 1, in which 0 means that the phases of the single cells are randomly distributed and 1 implies perfect synchrony (Gu and Yang, 2017; Meylahn, 2020). A limitation of the extended Kuramoto model is that the coupling strength would become infinite when the neuronal synchronization of the SCN is 100%. This problem is theoretical rather than practical: due to the differences in intrinsic characteristics of the neurons and noise in the system, perfect synchronization will never be reached (Maywood, 2020).

To recapitulate, with the 2-community Kuramoto model, we could determine the relationship between the coupling strength within neuronal subpopulations of the SCN and the interaction strength between the neuronal subpopulations, after we determined the phase coherence of SCN neurons in different experimental conditions. We found evidence that coupling strength within and between subpopulations correlates with photoperiod-induced changes in the phase relationship among neurons. In young mice, the SCN has a large adaptive capacity—as seen in the range of coupling strength—making them able to adapt to different photoperiods. With aging, the adaptive capacity of the SCN seems to be reduced. Aged animals seem to be unable to reach sufficient coupling strengths, which are necessary for correct encoding of short day-length in the SCN signal, making it more difficult for old mice to also behaviorally adapt to SP.

## Supplemental Material

sj-tif-1-jbr-10.1177_07487304231175191 – Supplemental material for Reduced Plasticity in Coupling Strength in the Aging SCN Clock as Revealed by Kuramoto Modeling Supplemental material, sj-tif-1-jbr-10.1177_07487304231175191 for Reduced Plasticity in Coupling Strength in the Aging SCN Clock as Revealed by Kuramoto Modeling by Anouk W. van Beurden, Janusz M. Meylahn, Stefan Achterhof, Renate Buijink, Anneke Olde Engberink, Stephan Michel, Johanna H. Meijer and Jos H. T. Rohling in Journal of Biological Rhythms
