# Supplementary figures and images for: Reduced Plasticity in Coupling Strength in the Aging SCN Clock as Revealed by Kuramoto Modeling
Source: J Biol Rhythms. 2023 Jun 16;38(5):461–75. doi: 10.1177/07487304231175191 (PMC10475211; doi:10.1177/07487304231175191)

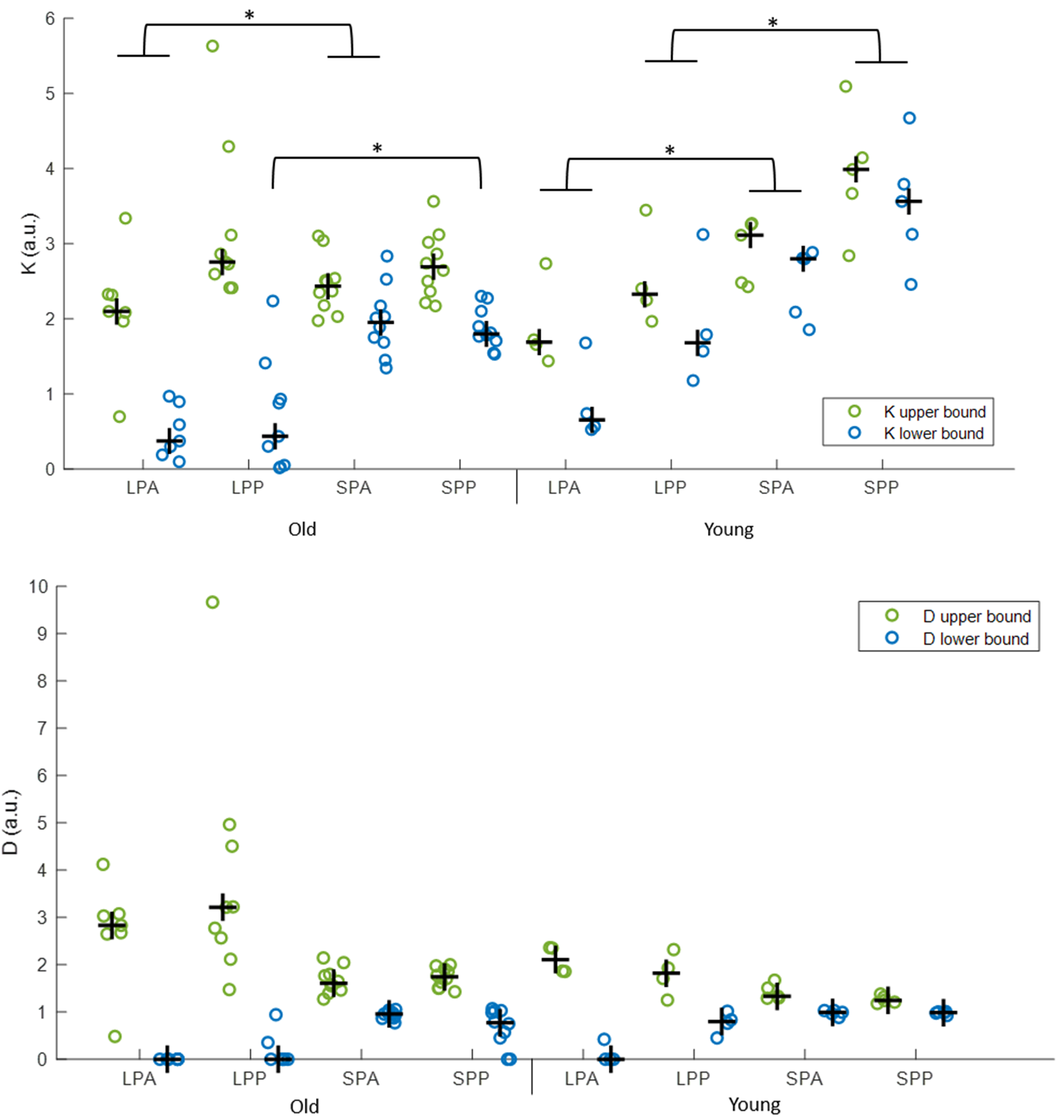

Supplement: sj-tif-1-jbr-10.1177_07487304231175191 – Supplemental material for Reduced Plasticity in Coupling Strength in the Aging SCN Clock as Revealed by Kuramoto Modeling [file sj-tif-1-jbr-10.1177_07487304231175191.tif]
